# Epigenetic control of *CD1D* expression as a mechanism of resistance to immune checkpoint therapy in poorly immunogenic melanomas

**DOI:** 10.3389/fimmu.2023.1152228

**Published:** 2023-04-03

**Authors:** Mona Meng Wang, Saara A. Koskela, Arfa Mehmood, Miriam Langguth, Eleftheria Maranou, Carlos R. Figueiredo

**Affiliations:** ^1^ Medical Immune Oncology Research Group (MIORG), Institute of Biomedicine, Faculty of Medicine, University of Turku, Turku, Finland; ^2^ Singapore National Eye Centre and Singapore Eye Research Institute, Singapore, Singapore; ^3^ InFLAMES Research Flagship Center, University of Turku, Turku, Finland

**Keywords:** immune checkpoint therapy, DNA methylation, β2M, CD1d, melanoma, MHC-I, SPI1, Anti-PD1

## Abstract

Immune Checkpoint Therapies (ICT) have revolutionized the treatment of metastatic melanoma. However, only a subset of patients reaches complete responses. Deficient β2-microglobulin (β2M) expression impacts antigen presentation to T cells, leading to ICT resistance. Here, we investigate alternative β2M-correlated biomarkers that associate with ICT resistance. We shortlisted immune biomarkers interacting with human β2M using the STRING database. Next, we profiled the transcriptomic expression of these biomarkers in association with clinical and survival outcomes in the melanoma GDC-TCGA-SKCM dataset and a collection of publicly available metastatic melanoma cohorts treated with ICT (anti-PD1). Epigenetic control of identified biomarkers was interrogated using the Illumina Human Methylation 450 dataset from the melanoma GDC-TCGA-SKCM study. We show that β2M associates with CD1d, CD1b, and FCGRT at the protein level. Co-expression and correlation profile of *B2M* with *CD1D*, *CD1B*, and *FCGRT* dissociates in melanoma patients following *B2M* expression loss. Lower *CD1D* expression is typically found in patients with poor survival outcomes from the GDC-TCGA-SKCM dataset, in patients not responding to anti-PD1 immunotherapies, and in a resistant anti-PD1 pre-clinical model. Immune cell abundance study reveals that *B2M* and *CD1D* are both enriched in tumor cells and dendritic cells from patients responding to anti-PD1 immunotherapies. These patients also show increased levels of natural killer T (NKT) cell signatures in the tumor microenvironment (TME). Methylation reactions in the TME of melanoma impact the expression of *B2M* and *SPI1*, which controls *CD1D* expression. These findings suggest that epigenetic changes in the TME of melanoma may impact β2M and CD1d-mediated functions, such as antigen presentation for T cells and NKT cells. Our hypothesis is grounded in comprehensive bioinformatic analyses of a large transcriptomic dataset from four clinical cohorts and mouse models. It will benefit from further development using well-established functional immune assays to support understanding the molecular processes leading to epigenetic control of β2M and CD1d. This research line may lead to the rational development of new combinatorial treatments for metastatic melanoma patients that poorly respond to ICT.

## 1 Introduction

Immune checkpoint therapies (ICT) can improve cancer patient survival by reinforcing the effector capabilities of antitumor T cells. Unfortunately, many patients are resistant to ICT. An important resistance mechanism to ICT is the lack of infiltration of antitumor immune cells, such as T lymphocytes, and higher frequency of immune-suppressive cells, such as myeloid-derived suppressor cells (MDSCs) (1). For that reason, these tumors are classified as immunologically “cold” tumors. Cold tumors are poorly immunogenic and often don’t respond to ICT. Immunologically “hot” tumors, however, have higher infiltration of antitumor T lymphocytes and are often more responsive to ICT (1).

The absence of tumor-infiltrating lymphocytes (TILs) and the presence of MDSCs in the tumor microenvironment (TME) can be the consequence of low tumor mutational burden (TMB). However, low TMB does not always translate into poor immunogenicity, and tumor-dependent resistance mechanisms may repress the generation of antitumor T cells leading to a lack of TILs (1). Resistance mechanisms associated with a deficient antigen presentation strongly impact the generation and expansion of tumor-specific T-cells. These mechanisms may be innate to the patients (refractory patients) or acquired (relapsed patients) after initial treatment (2, 3).

Tumors with innate or acquired resistance to ICT often develop features of cold tumors, such as lack of tumor-specific antigens, immune suppression, and low immune cell infiltration (1, 4, 5), governed by different cellular and molecular processes, including those impacting antigen presentation. For instance, the major histocompatibility molecules of class I and II (MHC-I and II), including their subcomponents, such as β2-microglobulin (β2M), encoded by the *B2M* gene in the human (6, 7). Evidence of ICT resistance shows that deficient β2M expression impacts the cell surface distribution of MHC-I, which impedes antigen presentation to the infiltrating antitumor cytotoxic T lymphocytes (CTLs) (2, 8).

However, MHC-I/II are not the only molecules responsible for presenting tumor antigens by antigen-presenting cells (APCs), and β2M has been previously described to interact with other MHC family molecules, such as CD1d, CD1b, and the neonatal Fc receptor FcRn (*FCGRT* gene) (9–12). Notably, the MHC-I-like protein CD1d is constitutively expressed by tumor and dendritic cells (DCs), responsible for the presentation of non-peptide antigens, such as lipids and small metabolites, to natural killer T (NKT) cells (13, 14). In addition, glycosphingolipids, such as the ganglioside GD3, have been demonstrated to contribute substantially to the immunogenicity of metastatic melanoma and are presented to NKT cells through CD1d by DCs, coordinating antitumor responses (15–19). More importantly, NKT cells have been recently described as a critical component of ICT-induced antitumor mechanism since these cells can also express immune checkpoint regulators, such as PD1 and others (20).

Taking into consideration recent findings on the epigenetic impact on β2M and associated faults in MHC-dependent peptide presentation leading to ICT resistance, we sought to investigate whether β2M deficiencies could also indicate deficiencies in other MHC family molecules, such as CD1d, and consequently, pointing a potential deficiency in the presentation of tumor glycoproteins and glycolipids as a contributing factor to ICT resistance. In this study, we revisited the tumor transcriptomic dataset of the skin cutaneous melanoma cohort (SKCM) of The Cancer Genome Atlas (TCGA) study (21), which is a part of The Genomic Data Commons (GDC). The GDC is a data-sharing platform developed by the National Cancer Institute (NCI) to support cancer research and hosts the TCGA project, which includes a large-scale collaborative effort to characterize the genomic and molecular features of multiple types of cancer, including melanoma. The TCGA datasets have been normalized and are ready to be used by integrated analysis platforms (such as Xena (22)) and re-published. In parallel, we revisited the publicly available transcriptomic datasets of different anti-PD1 metastatic melanoma cohorts (23–26) and concatenated the data using a TPM normalization protocol.

We evaluated whether the differential expression of *B2M* and its correlated genes (*CD1D*, *CD1B*, and *FCGRT*) is associated with poor responses to anti-PD1 in metastatic melanoma. We provide further evidence that epigenetic changes in the TME of melanoma tumors have a direct impact on *B2M* and an indirect impact on *CD1D* gene expression (through *SPI1*) but not on *CD1B* and *FCGRT* expression in melanoma patients from the TCGA dataset. This evidence suggests that methylation of *B2M* and *SPI1* might impact antigen presentation to T and NKT cells, respectively, ultimately suppressing local antitumor immune responses. These findings introduce new resistance mechanisms to anti-PD1 ICT and promote the rational development of new combinations targeting the epigenetic control of DCs/NKT activation to overcome resistance to anti-PD1 ICT in metastatic melanoma.

## 2 Justification of the hypothesis

### 2.1 β2M physically interacts with proteins associated with the presentation of self and non-self-glycolipids and predicts their differential clustering in metastatic melanoma

β2M is an essential molecular partner of the MHC-I complex (HLA class I in human and MHC-I in mouse). Such physical interaction is critical for the HLA/peptide complex formation for further priming (presentation) to antitumor T cells. Therefore, we sought to analyze what other clusters of molecules could also physically interact with β2M and potentially have a functional impact on cancer immunity and ICT outcomes. First, we performed a protein-protein interaction network functional enrichment analysis of β2M with neighboring proteins using Search Tool for the Retrieval of Interacting Genes/Proteins (STRING) (27). STRING is a bioinformatics database and web resource that provides information about protein-protein interactions, functional associations, and networks to evaluate the interaction of new potential biomarkers and systems immunology. STRING collects and integrates data from various sources, including experimental studies, computational predictions, and public databases. In this study, STRING is used to predict protein-protein interactions, including direct (physical) and indirect (functional) associations.

**Figure 1:**
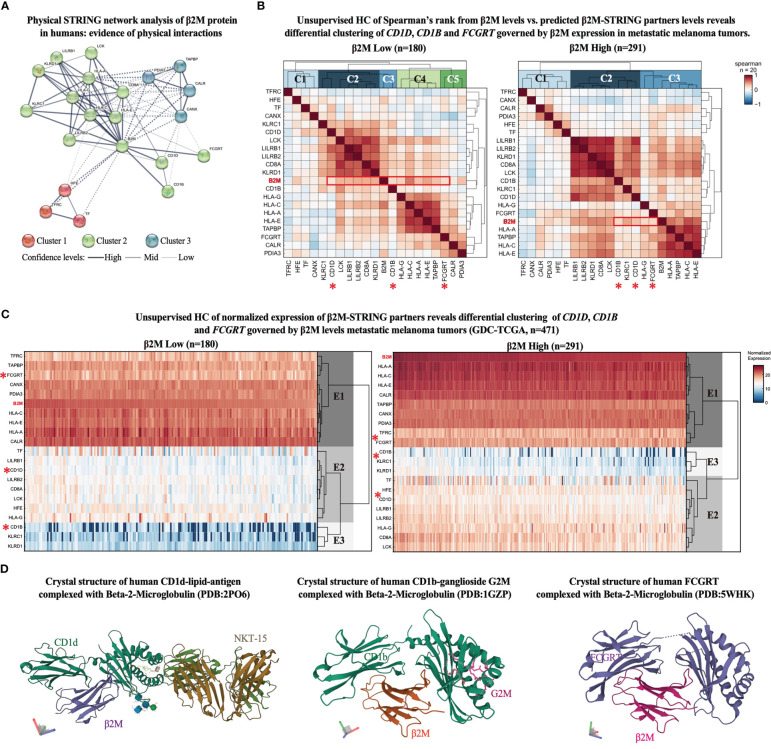
β2M expression associates with proteins responsible for antigen presentation of non-self-glycolipids in metastatic melanoma. **(A)** STRING network representation of human β2M-associated proteins. Edges indicate that the proteins are part of a physical complex distributed in the subnetwork. Line thickness indicates the strength of data support. The minimum required interaction score was set at 0.400. The k-means clustering method was used for network distribution to three clusters. Dotted lines represent edges between clusters. **(B)** Unsupervised hierarchical clustering (HC) of Spearman’s correlation scores between β2M expression levels and STRING-predicted β2M physical partners expression levels from metastatic melanoma samples from GDC-TCGA-SKCM study. Heatmaps represent positive correlation (up to 1, red), low correlation (close to 0, white), and negative correlation (up to -1, blue) scores in patients with low (left) and high (right) β2M expression. Expression cutoff: median of β2M expression in the cohort. **(C)** Unsupervised hierarchical clustering (HC) of STRING-predicted β2M physical partners. Columns are clustered using Euclidian/Ward metric/method. Paired color maps indicate raw expression values. **(D)** Crystal structure of β2M conjugated with CD1d (PDB: 2PO6), CD1b (PDB: 1GZP), and FCGRT (PDB: 5WHK) (28).

The functional enrichment analysis reveals that β2M has more interactions among HLA-related proteins than expected for a random set of proteins of the same size and degree distribution drawn from the human genome, suggesting that initially obtained clusters of proteins are at least partially biologically connected as a group. Expected connectivity enrichment was added to a network of 20 proteins. The k-means clustering method was applied to the network revealing three main clusters of proteins (red, green, and blue clusters) (**Figure 1A**).

As expected, the main β2M interacting cluster comprises proteins associated with antigen presentation *via* HLA class I to T cell receptors. Different HLA class I types are found in the cluster, as well as LCK (lymphocyte-specific protein tyrosine kinase), a protein kinase with essential roles in the function of mature T-cells once activated *via* antigen presentation by HLA class I molecules, LILRB (leukocyte immunoglobulin-like receptor B), which recognizes a broad spectrum of HLA-A, HLA-B, HLA-C, and HLA-G alleles, and natural killer (NK) cell antigens (KLRD1 or CD94) and KLRC1 (killer cell lectin-like receptor C1), which recognizes HLA class 1 in NK cells (29–31).

Notably, three other proteins showed high interaction scores with β2M, and despite having similar biological functions (HLA-associated antigen presentation), they cluster in an opposing edge of the network (**Figure 1A**). These proteins include CD1d, CD1b, and FCGRT, which characterize an edge in the network responsible for binding self and non-self-glycolipids and presenting them to NKT cells. Other edges include clusters 1 and 3, which roles are associated with iron recycling metabolism in non-canonical T cell activation and conjugation with APCs (32, 33), and calcium-dependent interaction of MHC class I with transporter associated with antigen processing (TAP) (34), respectively. These findings highlight β2M antigen-presenting functions other than those strictly associated with HLA genes to be further investigated for their potential antitumor immune responses in ICT outcomes. Therefore, we focused our further analyses solely on the proteins of the opposing edge of cluster 2, which is functionally associated with the antigen presentation of glycolipids (CD1d, CD1b, and FCGRT) (12, 19, 35).

**Figure 2:**
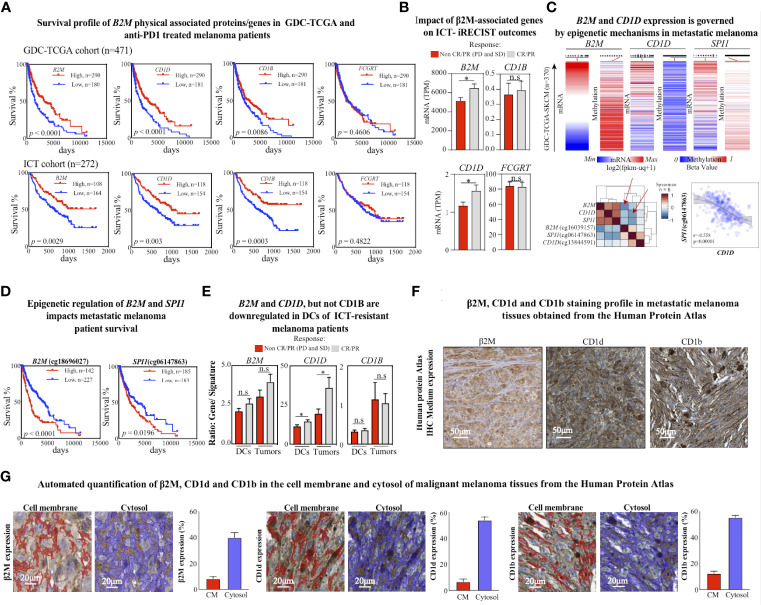
CD1d loss is associated with poor response to ICT. **(A)** Kaplan–Meier plots of overall survival for GDC-TCGA-SKCM metastatic melanoma cohort (upper panel) and anti-PD1 treated metastatic melanoma patients (bottom panel). The *B2M* low expression (blue lines) and high expression (red lines) groups were compared by the two-sided log-rank (Mantel-Cox) test. Cutoff curves were defined by their best fit in the Mantel-Cox test. **(B)** Expression levels of assigned genes (*B2M*, *CD1D*, *CD1B*, and *FCGRT*) in patients with metastatic melanoma treated with anti-PD1 ICT, responding or not responding to anti-PD1 treatment (pretreatment biopsies, n = 271). Two-tailed, unpaired t-test (* *p* < 0.05; n.s.: not significant). **(C)** Normalized mRNA expression and methylation beta levels obtained from Illumina Human Methylation 450 from GDC-TCGA-SKCM study. Methylation heatmaps were obtained from the promoter region of the corresponding gene. The lower left heatmap shows the euclidean distribution of Spearman’s rank correlation between normalized gene HTseq-FPKM-UQ scores and Illumina Human Methylation 450 beta values from the GDC-TCGA-SKCM study. Red arrows indicate significant negative correlation scores between *B2M* and cg cg18696027 methylation scores from *B2M*, and between *CD1D* and cg06147863 methylation scores from *SPI1*. The lower right plot shows the sigmoidal Pearson correlation curve between *SPI1* and the methylation in the *SPI1* promoter region. **(D)** Kaplan–Meier plots of overall survival for the GDC-TCGA-SKCM metastatic melanoma cohort for methylation levels of *B2M* and *SPI1* genes in their promoter regions. The low methylation (blue lines) and high methylation (red lines) groups were compared by the two-sided log-rank (Mantel-Cox) test. Cutoff curves were defined by their best fit in the Mantel-Cox test. **(E)** Comparison of gene expression (*B2M*, *CD1D*, and *CD1B*) and cell signature (DCs and melanoma cells) rations between metastatic melanoma responders and non-responders to ICT. Two-tailed, unpaired *t*-test (*p < 0.05; n.s.: not significant). Response defined by clinical criteria. **(F)** Immunohistochemistry representation of positive stainings for β2M, CD1d, and CD1b in malignant melanoma tissues were obtained from the Human Protein Atlas. β2M antibody code: CAB002572 and patient ID: 744. CD1d antibody code: CAB016107 and patient ID: 1369. CD1b antibody code: HPA021824 and patient ID: 1369. **(G)** Representative images of quantified tissue areas and automated quantification of β2M, CD1d, and CD1b expression in the cell membrane (in red) and cytosol (in blue) of malignant melanoma tissues from the Human Protein Atlas.

To evaluate whether the expression of *CD1D*, *CD1B*, and *FCGRT* is affected in a β2M-dependent manner in malignant melanoma, we first obtained the expression levels of all β2M-STRING-predicted interacting genes (n = 20) from the GDC-TCGA-SKCM study. Next, melanoma patients were sorted accordingly to *B2M* differential expression (high to low *B2M* expression). The median of *B2M* expression was used as a stratification cut-off. Then, we evaluated the correlation pattern of the expression levels of these genes across patients with high and low *B2M* expression and performed an unsupervised hierarchical clustering (HC) of the correlation scores, which indicates the clustering pattern of patients with high and low *B2M* levels (clusters C1, C2, C3, etc.). As observed in the correlation matrix of **Figure 2B**, different *B2M*-associated markers show a range of correlation scores from -1 to +1, including HLA and NK genes with high correlation scores with different markers. These scores are useful to provide sufficient complexity to the matrix for further unsupervised clustering analysis, of which purpose is not to evaluate markers with higher or lower correlation but ultimately to observe how the cluster identity of *CD1D*, *CD1B*, and *FCGRT* fluctuates in the context of patients expressing high and low *B2M*, providing further insight on whether antigen-presentation of glycolipids governed by these genes (12, 19, 35) could be impacted by *B2M* differential expression in melanoma.

Patients with high *B2M* expression have a correlation diversity represented by clusters C1, C2, and C3 (**Figure 1B**, right correlation heatmap), and patients with *B2M* loss have higher correlation diversity, represented by clusters C1, C2, C3, C4, and C5 (**Figure 1B**, left correlation heatmap). We observed that gene *B2M* correlation scores with *CD1D*, *CD1B*, and *FCGRT* is located in different clusters between patients with high and low *B2M* expression. *B2M* and *CD1B* correlation score ranks in cluster C2 from patients with high *B2M* expression, as opposed to cluster C3, from patients with low *B2M* expression.

Similarly, *B2M* and *FCGRT* correlation scores shift from cluster C3 (patients with high *B2M* expression) to cluster C5 (patients with low *B2M* expression). *CD1D*, however, remains in the correlation cluster C2 in both groups. We also performed an HC analysis of the expression scores of these genes across patients with high and low *B2M* expression. Although *CD1D*, *CD1B*, and *FCGRT* are distributed in the same expression clusters (E1, E2, and E3), there is an evident dispersion of their expression score in the heredogram cluster, which can be explained by the downregulation of these genes in patients with *B2M* expression loss (**Figure 1C**). Since *CD1D*, *CD1B*, and *FCGRT* distribution across correlation (C) and expression (E) clusters are dispersed following *B2M* differential expression, these findings indicate that the expression levels of these genes and their association may be impacted in the same biological context governing *B2M* differential expression in melanoma.

Further representation of the molecular interaction of β2M with CD1d, CD1b, and FCGRT was obtained from the Protein Data Bank (PDB), in which the crystal structure of β2M with CD1d, CD1b, and FCGRT was previously determined (**Figure 1D**). Altogether, findings suggest that loss of β2M expression may not only play an important role in MHC-I deficiency as a mechanism of immunosuppression and ICT resistance (2, 8), but also impact other important β2M-dependent biological processes, such as the ones related to non-classical MHC proteins (CD1d, CD1b, and FCGRT) are potentially impacted by β2M loss and may contribute to ICT resistance.

### 2.2 CD1d downregulation is associated with poor metastatic melanoma outcome and resistance to ICT

Since β2M is an essential component of MHC-I antigen presentation to CD8+ T cells in the context of tumor immunity (36), and downregulation of HLA-I has been associated with deficient expression of β2M in ICT-resistant metastatic melanoma patients (8), we sought to evaluate whether CD1d, CD1b, and FCGRT, could also be associated with β2M-dependent ICT resistance. An expanded analysis of the GDC-TCGA-SKCM study confirmed that loss of *B2M* is significantly associated with poor survival outcomes (**Figure 2A**, upper panel). Then, we normalized and combined four transcriptomic publicly available cohorts of metastatic melanoma patients receiving anti-PD1 therapies and evaluated the survival responses regarding *B2M* differential expression. We confirmed that *B2M* loss is also strongly associated with reduced survival in patients treated with anti-PD1 therapies (**Figure 2A**, lower panel). Notably, a similar pattern of survival outcome was also observed for *CD1D* and *CD1B* expression profiles but not for *FCGRT* (**Figure 2A**). These findings suggest that loss of *B2M* may not only impact the biological processes related to the priming of CD8+T cells but also impact other β2M-dependent biological processes, such as CD1-associated antigen presentation, contributing to ICT resistance.

We performed an alternative analysis of the differential expression of *CD1D*, *CD1B*, and *FCGRT* between responders (CR and PR) and non-responders (SD and PD) to ICT outcome as previously described (37). Notably, only *CD1D* and *B2M* differential expression significantly predict response to anti-PD1 following the clinical response criteria (**Figure 2B**). These findings reinforce a potential *CD1D*-dependent role associated with *B2M* loss in ICT-driven resistance rather than solely regulating the priming of CD8+ T cells through MHC class-I. Indeed, previous evidence suggests that *B2M* is important to provide physical stability to CD1d protein and that downregulation of *B2M* would drive the CD1d degradation (38).

Similar survival outcome predictions by *B2M* and *CD1D* suggest that differential expression of these genes might be governed by a shared mechanism of gene expression control rather than solely a protein-protein relationship. Epigenetic changes have been suggested as a general mechanism of gene expression control in the context of the ICT resistance (2), such as DNA methylation, which recruits proteins to repress gene expression or inhibits the binding of a transcription factor to the DNA (39).

Studies from the past decade have shown that epigenetic mechanisms largely influence the fate of immune cell differentiation in the cancer (40). Moreover, previous evidence shows that DNA methylation predicts response to ICT in metastatic melanomas (41). Therefore, we sought to evaluate whether *B2M* and *CD1D* gene expression is governed by epigenetic changes associated with DNA methylation in the tumor microenvironment of melanoma. We revisited the methylome profile of metastatic cutaneous melanoma patients from the GDC-TCGA study. We observed that the expression of *B2M* is negatively correlated with increased methylation of the promoter region cg18696027 (**Figure 2C**, upper panel), which is also significantly associated with a poor survival response to ICT (**Figure 2D**, left).

Interestingly, *CD1D* differential expression control is not correlated to differential methylation in the promoter region of *CD1D* (**Figure 2C**, upper panel). However, we found that the differential expression of the transcription regulator of *CD1D*, the transcription factor *SPI1*, is negatively correlated with methylation levels at the promoter region (**Figure 2C**, upper panel), which is also significantly associated with a poor survival response to ICT (**Figure 2D**, right). These findings suggest that epigenetic changes in the TME might directly impact the expression of *B2M* and indirectly impact the expression of *CD1D* through *SPI1* regulation.

Next, we evaluated whether *B2M*, *CD1D*, and *CD1B* differential expression are impacted at the tumor cells or DCs levels in the TME. Using previously validated immune gene signatures from bulk RNA datasets to estimate immune cell abundances (42), we found that *B2M* and *CD1D* but not *CD1B* were significantly enriched on both tumor and DCs cells from patients responding to ICT (**Figure 2E**). Evidence of the expression profile of β2M, CD1d, and CD1b in melanoma tissues can be found in the Human Protein Atlas collection. Representative regions of positively stained melanoma tissues are shown, where the cell nuclei are labeled in blue color, and the protein is stained with brown color (**Figure 2F**). According to the atlas annotation, β2M, CD1d, and CD1b proteins are predominantly expressed in the cell cytoplasm and membrane. The expression levels of these markers were then quantified as described previously (43). As opposed to the cell membrane region, β2M, CD1d, and CD1b were mostly expressed in the cytosol of malignant melanoma tissues (**Figure 2G**). The frequencies of positively stained areas of the cell membrane and cytosol within the tumor tissue are as follows: β2M 8.2% (cell membrane) and 39.84% (cytosol); CD1d 6.72% (cell membrane) and 53.83% (cytosol); and CD1b 12.39% (cell membrane) and 55.18% (cytosol) (**Figure 2G**). These findings suggest that the expression profile of these genes in cellular components of the TME may be a critical step to engage innate cellular mechanisms of response to ICT, with potential further consequences, such as NKT cell activation.

**Figure 3:**
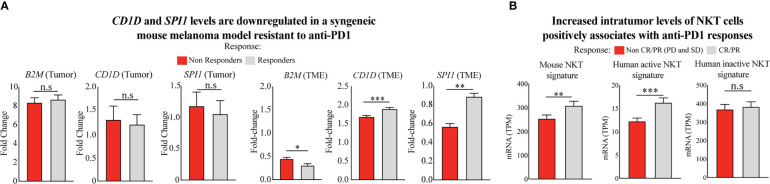
Poor response to ICT given is associated with reduced levels of intratumor NKT cells. **(A)** Comparison of gene expression of *CD1D* (*CD1d1* in mouse) and *SPI1* in the TME of anti-PD1 responsive and non-responsive mouse models (44). Two-tailed, unpaired *t*-test (***p < 0.0001; **p < 0.001; *p < 0.05; n.s., not significant). **(B)** Comparison of NKT cell levels using multigene NKT signature validated pairwise by the mouse model in bulk and single-cell datasets (45) and human active (stimulated) and inactive (unstimulated) NKT cell signature (46). Differential signature expression is shown between metastatic melanoma patients responding and not responding to anti-PD1 therapy. Patient samples were included from the integrated cohort of human melanoma patients receiving mainly anti-PD1 immunotherapies (23–26). Responders and non-responders to ICT were stratified using RECIST V1.1 (47) and iRECIST (48) criteria. Two-tailed, unpaired *t*-test (***p < 0.0001; **p< 0.001; n.s., not significant).

The differential expression of *CD1D* and *SPI1* genes and their impact on ICT outcome was further confirmed in the microarray dataset of tumors, and TME obtained from mice with resistance or response to anti-PD1 ICT, as previously described (44). From this dataset, we observed no differences in the expression profile of these genes within the tumors of mice with and without resistance to anti-PD1 ICT (**Figure 3A**, left). However, *CD1D* and *SPI1*, but not *B2M*, are significantly downregulated in the TME of anti-PD1 resistant mice (**Figure 3A**, right). Moreover, in the TME level, *B2M* shows a slight increase in the anti-PD1 resistant group, which does not support the results obtained from the human dataset, potentially given to natural differences existing cross-species (mouse and human), at least in the level of *B2M*.

CD1d expression is crucial for presenting glycoprotein and glycolipid antigens to NKT cells (19). As a result, NKT cells become activated and exhibit enhanced survival and proliferative capacity in response to α-GalCer-mediated activation (49). Therefore, we interrogated the transcriptome of tumor biopsies from the integrated cohort of human melanoma patients before receiving anti-PD1 immunotherapies (23–26) to evaluate potential differences in intratumor NKT cell levels. We used the metagene expression values of the NKT cell signature to perform this analysis as previously described (45). First, we used an NKT cell signature validated in bulk and single-cell RNA datasets from healthy mouse tissues, including skin, lymph nodes, and lungs (45), the preferential sites for metastatic melanoma development. When interrogating the expression levels of this multigene signature in our combined cohort of anti-PD1 treated melanoma patients, we found that anti-PD1 responsive patients show significantly higher intratumor levels of NKT cell signature instead of non-responsive patients (**Figure 3B**, left). Since recent evidence has shown that innate immune cells have shared developmental programs in mice and humans (50), we also performed a similar analysis using human multigene signatures from stimulated and unstimulated human NKT cells described elsewhere (46), and with shared gene composition found in the mouse signature. Interestingly, we observed that only the multigene signature from human NKT cells previously stimulated and with an active phenotype are upregulated in patients responding to anti-PD1 therapy (**Figure 3B**, center), as opposed to the signature from unstimulated-inactive NKT cells (**Figure 3B**, right). These findings suggest that NKT cells with an active phenotype may display an important role following anti-PD1 therapies in patients with malignant melanomas, given their known antitumor properties, in which activation and frequency levels might be controlled by epigenetic changes regulating *B2M*, *SPI1*, and *CD1D* expression in anti-PD1 resistant tumors.

## 3 Discussion

**Figure 4:**
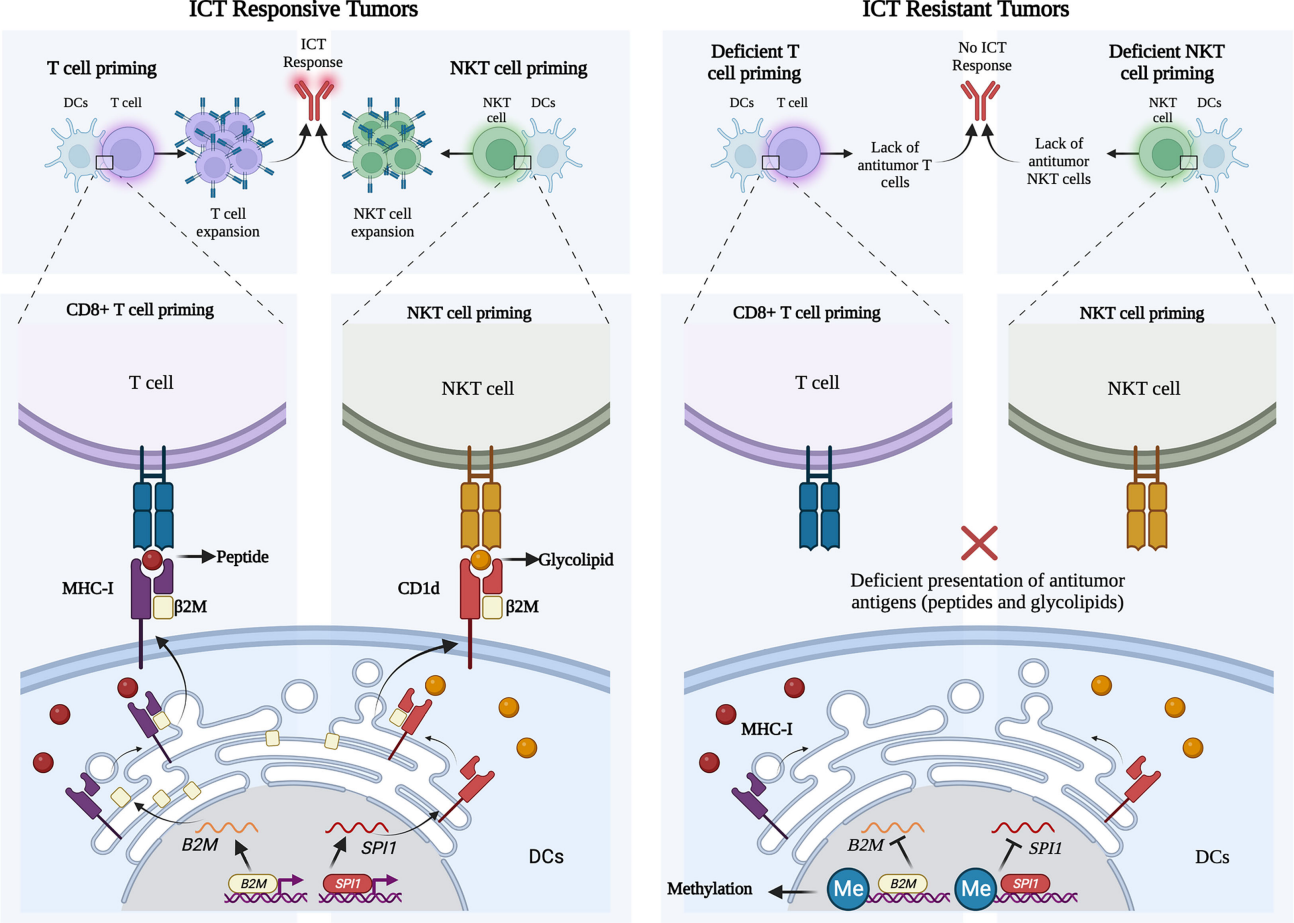
Schematic representation of *B2M* and *CD1D* expression profile in ICT responsive and resistant melanoma tumors. Left panel: Successful priming of T cells and NKT cells by dendritic cells with the physiological engagement of β2M with MHC and CD1d, respectively. Right panel: Deficient priming of T cells and NKT cells by dendritic cells given to methylation of promoter regions of *B2M* and *SPI1*, respectively, in patients not responding to ICT.

In this article, we revisited the molecular interaction of β2M with CD1d, CD1b, and FCGRT and its implication for ICT response. Evidence for a new mechanism of ICT resistance is provided by differential expression of β2M/CD1d axis dependent on epigenetic alterations in the TME of metastatic melanoma, which is significantly associated with poor outcomes of metastatic melanoma patients receiving anti-PD1 immunotherapies (**Figure 4**).

Existing evidence of physical interactions between β2M with CD1d, CD1b, and FCGRT suggests that these interactions may play an important functional role in the context of APCs, supported by the fact that β2M deficiencies significantly reduce the tumor antigen presentation *via* MHC-I, resulting in poor ICT outcome (2). Indeed, In the absence of β2M protein, CD1d can be transported to the cell surface independently, and glycosylation patterns of CD1d drive its rapid degradation to an immature glycoprotein state (38).

Antigen presentation is a critical stage in eliciting anti-tumor responses by ICT (51), which is not restricted to tumor peptides presented *via* MHC molecules to T cells, but includes antigen presentation *via* MHC-associated molecules, such as β2M and CD1d, which presents non-peptide (glycolipid) antigens, such as α-galactosylceramide (αGalCer), to antitumor NKT cells (52, 53). β2M deficiency causes downregulation of MHC-I expression and deficient CD1d-dependent NKT cells leading to complex immunodeficiency clinically (54). We hypothesize that the presentation of antigens to NKT cells, which depends on β2M/CD1d, is critically important for ICT clinical benefit.

Supporting this hypothesis, studies have shown that activation of NKT cells can lead to improved outcomes in mice and patients receiving ICT, mainly manifested by the increase of IL-2 (Interleukin 2) and IL-12 (Interleukin 12) by NKT cells upon CD1d stimulation by αGC in APCs, reinvigorating exhausted CD8 T cells in synergism with anti-PD1 therapies in tumor-bearing mice and patients with cancer (55). Indeed, NKT effector functions comprise abundant cytokine released upon activation (56), which in turn, reinvigorates other cytotoxic immune cells and effector responses of CD8+ T cell (57, 58). PMA/ionomycin stimulated NKT cells (as the ones profiled using human signatures in the anti-PD1 cohort of this study) previously presented to α-GalCer antigen through DCs-expressing CD1d, have been described to enhance the expression of IFN-γ, which ultimately impacts cancer immune microenvironment by increasing iNOS+CD206- M1 macrophage levels for melanoma control growth (59).

As a direct target of anti-PD1 therapy, little information is available about exhausted NKT cells in the tumor microenvironment. However, recent studies using the E0771 breast cancer and B16 melanoma models have found that in the later cancer stages, NKT cells have impaired cytotoxic capacities manifesting an exhausted phenotype (60). In addition, increased levels of total NKT cells have been observed in biopsies from anti-PD1 responding patients (61, 62). Therefore, molecular mechanisms driving NKT generation and proliferation, such as the antigen presentation of glycolipids and glycoproteins, should be characterized in melanoma biopsies to orient the development of new combinatory therapies.

*CD1D* differential expression is associated with metastatic melanoma patient survival outcomes from our analysis. Consistent with our finding, downregulation of *CD1D* has been documented in many cancer types, such as breast (63) and cervical carcinoma (64), and was associated with poor survival outcomes (65). Using non-small cell lung cancer (NSCLC) cells as a model, the downregulation of *CD1D* expression demonstrated limited NKT activities achieved by epigenetic modifications in those tumors (66, 67).

Indeed, cancer-specific changes in DNA methylation and histone acetylation result in pro-tumorigenic functions and repression of tumor suppressor genes (68) and have been associated with ICT resistance (2). Methylation levels of the gene *SPI1* promoter region indirectly controlled *CD1D* gene expression. There are currently no reported studies investigating the relationship between *SPI1* gene expression and resistance to ICT in melanoma., but this gene is downregulated and methylated in more than 70% of lymphoma patients (69).

Therefore, epigenetic therapeutic strategies that have been observed to improve ICT and modulate NKT cell responses, such as HDAC inhibitors, might mechanistically promote the increase in *B2M* and *CD1D* expression to support antigen presentation and generation of T cells and NKT cells (70–72).

The strength of our study is that it includes a robust combination of four transcriptomic datasets of anti-PD1 immunotherapies, compared with the TCGA dataset and a model of metastatic melanoma resistant to anti-PD1 therapy. However, a limitation of our study is the absence of *in vitro* and *ex vivo* validation using well-established functional immune assays, which are now considered to validate these findings and develop new therapeutic opportunities by our group.

In conclusion, this study conceptualizes the importance of further investigating therapeutic strategies to restore *CD1D* expression in metastatic melanoma by targeting epigenetic modulators in the TME. Restored expression of *CD1D* can be further compared with the effector functions of NKT cells and survival outcomes in preclinical and clinical research. Moreover, our findings point to new horizons for the rational development of combinatory approaches that block epigenetic mechanisms of suppression in the TME to restore DCs-dependent antigen presentation to NKT cells, which can lead to better responses to anti-PD1 immunotherapies in metastatic melanomas.

## 4 Materials and methods

### 4.1 Human cohort RNA-seq data sets and analysis

The mRNA expression and survival data of The Cancer Genome Atlas SKCM GDC dataset were downloaded from the Xena Functional Genomics Explorer of the University of California, Santa Cruz (https://xenabrowser.net/datapages/and https://xenabrowser.net/heatmap/) (73). The Genomic Data Commons (GDC) legacy archive obtained samples with methylation. The methylation sites tested are listed as follows: *B2M* (cg18696027), *SPI1* (cg06147863), and *CD1D* (cg13844591). Generated data were extracted in tab‐separated values (TSV) format. RNA-seq datasets from all patients receiving immune checkpoint therapies were collected from anti-PD1 Riaz cohort (23) (access numbers: GSE91061 and SRP094781), anti-PD1 and anti-PD1/anti-CTLA4 Gide cohort (25) (access number: PRJEB23709), anti-PD1 Hugo cohort (26) (access number: GSE78220) and anti-PD1 Liu cohort (24) (access number: phs000452.v3.p1), available at Gene Expression Omnibus (GEO), Sequence Read Archive (SRA), European Nucleotide Archive (ENA) and database of Genotypes and Phenotypes (dbGaP). The downloaded SRA files were converted into FASTQ files using SRA-Toolkit (2.11.0). The quality of the sequenced reads was observed using FastQC (v0.11.9) tool (74). Transcript per Million (TPM) values were quantified using the human reference genome (GRCh38) and Kallisto (v0.48.0) (75). The transcript per million (TPM) values from the Liu dataset are also available in the Supplementary Data of the original publication (24). The TPM values of all the ICT cohorts were adjusted for batch correction using the R package ComBat-seq (76). Since it is our goal to measure the efficacy of responses based on the tumoricidal potential of effector immune cells, such as NKT cells, as described elsewhere (37), in our study, we consider Overall Response Rates (ORR) and not disease control (DC), to define the group of patients responding to the tumoricidal effects of ICT, and not cytostatic effects, accordingly to pre-established measurement guidelines (77). For this purpose, the response criteria include partial (PR) or complete response (CR) to patients responding to ICT, and stable disease (SD) and progressive disease (PD) to patients not responding to ICT. Riaz (23), Gide (25) and Liu (24) cohorts define response patterns using Response Evaluation Criteria for Solid Tumors version 1.1 (RECIST V1.1) (47), and Hugo cohort (26) uses the Immune-Related Response Evaluation Criteria for Solid Tumors (iRECIST) (48). SD patients from irRECIST cohorts (Hugo et al) were not included in this study due to the absence of transcriptomic data from these patients.

NKT cell expression was compared in responder and non-responder groups from the integrated ICT cohorts (23–26). Mouse NKT multigene signature was identified in a preclinical mouse model by screening the top 30 upregulated genes highly expressed in NKT cells enriched from the skin, lymph nodes, and lungs, which was validated in bulk and single-cell datasets (45). Human active and inactive NKT signatures were identified by analyzing matched signature components presented in a single-cell signature from stimulated and unstimulated human NKT cells (46), also present in the mouse signature. Overall survival was evaluated with survival and survminer package using a quartile range from 0.25 to 0.75 that obtained the minimum p-value. Survival was visualized as Kaplan-Meier (Log-rank) plots.

### 4.2 RNA Microarray analysis from preclinical ICT resistance melanoma model

Microarray data of tumor cells and TME from responding and non-responding mice to ICT were retrieved from the GEO database (number GSE122222 (44). The raw mRNA expression data were firstly normalized using a list of housekeeping genes (*CHMP2A, PSMB2, PSMB4, REEP5, SNRPD3, VCP, VPS29*) as previously described (78). The transcriptomic expression of tumor cells and TME were then calculated by averaging the normalized value by each housekeeping gene. Fold change analysis was performed as previously described (79).

### 4.3 Immunohistochemistry analysis

The Human Protein Atlas database provides the immunohistochemistry (IHC) protein staining for 15287 genes in 20 cancer types (80). For cutaneous melanoma tissues, the fraction of samples with protein expression levels is provided with 3, 3’-diaminobenzidine (DAB) staining in amelanotic areas. Scale bars indicate 50 and 20 μm in length. The protein expression level of β2M, CD1d, and CD1b in the cell membrane and cytosol were quantified using an automated machine learning quantification method based on marker intensity, color, density, and object size and circularity, as described previously (43). The frequency (%) of positive expression relative to the total area is represented by bar plots indicating Means ± Standard Error of Mean (SEM) from five independent quantified tissue areas from melanoma tissues.

### 4.4 Quantifications and statistical analysis

Graphpad prism 9.0 software was used for statistical analysis. The Shapiro-Wilk normality test was performed for all data sets to guide statistical tests. Unpaired two-tailed Mann-Whitney U test (non-parametric) was used with non-normally distributed samples group analysis. Non-parametric two-tailed Spearman’s correlation test was applied to non-normally distributed samples, and Pearson’s correlation test was applied to normally distributed samples. Graphpad prism 9.0 was used to generate all Kaplan Meier survival plots. The survival log-rank test was used to evaluate the significance of survival curves providing X^2^ (chi-squared) and p values. Differences were considered significant when *p <* 0.05. The two-tailed, unpaired *t*-test is used to determine whether there is a significant gene expression difference between independent groups. Z score transformation of transcriptomic data and heat cluster data analysis was performed using InstantClue (81).

## Data availability statement

The original contributions and data availability presented in the study are publicly available datasets and are referenced in the article. Further inquiries can be directed to the corresponding author.

## Author contributions

MW: Conceptualization, data curation, formal analysis, investigation, visualization, methodology, writing–review, and editing. SK: Data curation, software, formal analysis, validation, investigation, methodology, writing–review, and editing. AM: Data curation, software, formal analysis, and validation. ML and EM: writing–review and editing. CF: Conceptualization, data curation, formal analysis, supervision, funding acquisition, validation, methodology, writing-original draft, supervising the research. All authors contributed to the article and approved the submitted version.
